# A computational model on the goldfish Mauthner cell

**DOI:** 10.1186/1471-2202-15-S1-P97

**Published:** 2014-07-21

**Authors:** Tuomo Mäki-Marttunen, Violeta Medan

**Affiliations:** 1 Departamento de Fisiología, Biología Molecular y Celular, Universidad de Buenos Aires, Argentina; 2 Department of Signal Processing, Tampere University of Technology, Finland; 3 Instituto de Fisiología, Biología Molecular y Neurociencias, Universidad de Buenos Aires - CONICET, Argentina

## 

Integration of multimodal information is of key importance to generate adaptive behavior. However, our understanding of how multimodal integration is implemented at the dendritic level is still scant. We address this question in the Mauthner-cell (M-cell), the “decision making element” [1] of the startle escape network of goldfish. The M-cell has two main aspiny dendritic branches arising from the soma, the lateral dendrite and the ventral dendrite, where the former receives auditory input [2] and the latter input from the visual system [3]. Both dendrites are amenable to intracellular recording in vivo, which offers the opportunity to study whether the propagation of auditory and visual signals is similar, or if different filtering properties are implemented in each dendrite. To describe the cell behavior, we use a Hodgkin-Huxley type of model [4,5] for the spike initiation zone, combined with a realistic dendritic morphology reconstructed from intracellular staining images. We fit the model parameters to intracellular recordings, where the cell is stimulated with square and ramp pulses injected at the soma, and responses are measured at a fixed location in the proximal lateral dendrite. The obtained model provides a simulation framework for studying the signal propagation along the cell dendrites and soma. Simulations of impulse decay along passive dendrites in orthodromic and antidromic directions are compared with the corresponding experimental observations. In the orthodromic direction, the model correctly predicts that the spatial decay is larger on the ventral than on the lateral dendrite, due to the differences in the dendrite diameters. However, recordings of signals propagating antidromically in the ventral dendrite show smaller spatial decay than those in the lateral dendrite, opposite to the model predictions. Our preliminary results suggest that passive dendrites alone are not enough to explain the experimentally observed spatial decay in the two different directions. By contrast, we found that the application of voltage-gated ion channels to the model of the ventral dendrite, even with very small maximal conductances, could lead to correctly reproducing the observed signal propagation properties. Although traditionally the soma and dendrites of the M-cell are considered to be purely passive, voltage-dependent conductances have been observed on the lateral dendrite [6]. To make sure the results are not an artifact of a certain complex dendrite morphology, we confirm our findings using an approximative, simple dendritic morphology. Our results highlight 1) the importance of including realistic morphology on modeling studies of neuron behavior; 2) the possibility of specialization of the dendritic arbors within the same neuron depending on the input they receive; and 3) the possibility of computationally examining the existence of active conductances in neurons that do not produce dendritic spikes. The implications of the existence of active dendritic compartments for the cell functioning are discussed in the context of the integration capabilities of single neurons.
